# Comparative Genomics of *Helicobacter pylori* Strains of China Associated with Different Clinical Outcome

**DOI:** 10.1371/journal.pone.0038528

**Published:** 2012-06-06

**Authors:** Yuanhai You, Lihua He, Maojun Zhang, Jianying Fu, Yixin Gu, Binghua Zhang, Xiaoxia Tao, Jianzhong Zhang

**Affiliations:** State Key Laboratory for Infectious Disease Prevention and Control, National Institute for Communicable Disease Control and Prevention, Chinese Center for Disease Control and Prevention, Beijing, China; J. Craig Venter Institute, United States of America

## Abstract

In this study, a whole-genome CombiMatrix Custom oligonucleotide tiling microarray with 90000 probes covering six sequenced *Helicobacter pylori* (*H. pylori*) genomes was designed. This microarray was used to compare the genomic profiles of eight unsequenced strains isolated from patients with different gastroduodenal diseases in Heilongjiang province of China. Since significant genomic variation was found among these strains, an additional 76 *H. pylori* strains associated with different clinical outcomes were isolated from various provinces of China. These strains were tested by polymerase chain reaction to demonstrate this distinction. We identified several highly variable regions in strains associated with gastritis, gastric ulceration, and gastric cancer. These regions are associated with genes involved in the bacterial type I, type II, and type III R-M systems. They were also associated with the *vir*B gene, which lies on the well-studied cag pathogenic island. While previous studies have reported on the diverse genetic characterization of this pathogenic island, in this study, we find that it is conserved in all strains tested by microarray. Moreover, a number of genes involved in the type IV secretion system, which is related to horizontal DNA transfer between *H. pylori* strains, were identified in the comparative analysis of the strain-specific genes. These findings may provide insight into new biomarkers for the prediction of gastric diseases.

## Introduction

*Helicobacter pylori* (*H. pylori*) is a Gram-negative bacterial pathogen responsible for human gastric diseases, including gastritis and peptic ulcers. It is also a major risk factor in the development of gastric cancer [1]–[3]. In 1994, the World Health Organization listed *H. pylori* as a class I carcinogen.

Previous studies have shown that *H. pylori* has a high rate of homologous gene recombination. Phylogenetic analysis have subdivided *H. pylori* into distinct biogeographic populations and subpopulations with specific geographical distributions that reflect ancient human migrations. The studies also indicated that the East Asian type (hpEastAsia) is classified into at least three subtypes: East Asian (hspEAsia), Pacific (hspMaori) and native American (hspAmerind). The East Asia subtype (hspEAsia) may be related to the high incidence of gastric cancer in East Asia [4]–[7]. A more recent study demonstrate the East Asian group appear to differ greatly from the European group in electron transfer and redox reactions, which suggest a model of adaptive evolution and selection through proteome diversification and modulation of translational fidelity [8].

A number of studies have focused on the *cag*- pathogenicity island encoded virulence apparatus that may play a critical role in *H. pylori* pathogenesis [9]–[11]. Importantly, almost all isolates from East Asia harbor the *cag*-PAI island. A few studies have shown that there are specific sequence characteristics shared among these islands in the various strains, which are responsible for causing different diseases [12]–[14]. This suggests that genome-wide analyses in various worldwide strains, especially for strains found in East Asia, are necessary. To date, there are thirty-two completed genomes and four draft genomes available on GenBank [15]–. Genome sizes among clinical isolates of *H. pylori* vary considerably, with some showing differences up to 25% [28], [29]. Comparative genomic profiling, using microarrays designed to cover entire genomes, is one strategy that can be used to obtain information about the variability between strains isolated from different patients and locations as well as to indicate horizontal gene transfer. Previously available commercial chips cover only one or two *H. pylori* genomes [30]–[37]. With more *H. pylori* genome sequences publicly available, it is possible to design high-density microarrays covering all of these fully sequenced genomes. During this study, an additional twenty-six strains associated with different clinical outcomes of various phylogeographic lineages were completely sequenced in the last two years. Additionally, several strains from other countries are also now undergoing sequencing. Here we describe the design and use of a high-density oligonucleotide microarray covering six sequenced *H. pylori* genomes. The performance of this microarray is evaluated, and we illustrate its utility for the hybridization of genomic DNA in order to compare eight uncharacterized *H. pylori* strains to the six established strains. We use this microarray to identify variable genomic regions among *H. pylori* strains isolated from patients with different gastroduodenal diseases in a Chinese patient population.

**Table 1:** Characteristics of the 84 *H. pylori* strains studied.

Origin	Clinical Diagnosis	No. of strains
Heilongjiang (n = 23)	GC	12
	DU	3
	GU	3
	AG	3
	SG	2
Shandong (n = 12)	GC	12
Hubei (n = 10)	FD	6
	GU	2
	DU	2
Xi’an (n = 11)	SG	7
	DU	1
	GDU	3
Yunnan (n = 10)	SG	5
	GU	3
	DU	2
Jiangxi (n = 18)	SG	9
	GC	9

Note: GC, gastric cancer. GU, gastric ulcer. AG, atrophic gastritis, DU, duodenal ulcer, GDU, gastroduodenal ulcer. FD, functional dyspepsia. SG, non-atrophic gastritis.

## Materials and Methods

### H. Pylori Strains

*H. pylori* is a well-known pathogen with much genetic variation; both host and bacterial factors may contribute to this fact. Strains isolated from different countries, different geographies, and even different families show genomic diversity [38]. Therefore, from a genomic viewpoint, strain selection has been a critical issue for investigation of the pathogenic mechanism. In this study, we chose eight strains for microarray analysis. This included isolates from 2 patients with chronic superficial gastritis (HLJ220,HLJ215), 2 patients with atrophic gastritis (HLJ193,HLJ256), 2 patients with gastric ulcers (HLJ271,HLJ253), and 2 patients with gastric cancer (HLJ038,HLJ005). The strain numbers were encoded by our lab, Department of Diagnosis for Communicable Diseases, National Institute for Communicable Disease Control and Prevention, China CDC. They were all isolated from Heilongjiang province of China, which reports a high incidence rate of gastric cancer. We used PCR for investigating an additional 76 strains of disease specific genes, which were isolated from seven Chinese provinces. These included thirty-one gastric cancer strains (GC), six gastric ulcer strains (GU), one atrophic gastritis strain (AG), eight duodenal ulcer strains (DU), three gastroduodenal ulcer strains (GDU), six functional dyspesia strains (FD), and twenty-one non-atrophic gastritis strains (SG). Background information describing these strains is briefly listed in table 1.

**Table 2:** General features of the sequenced *H. pylori* genomes selected for microarray probe design.

strain	ACCESSION	length	origin	Clinical diagnosis
26695	AE000511	1667867	UK	Gastritis
J99	AE001439	1643831	USA	Duodenal ulcer
HPAG1	CP000241	1596366	Sweden	Atrophic gastritis
P12	CP001217	1673813	German	Duodenal ulcer
G27	CP001173	1652982	Italy, Tuscany	No known disease
Shi470	CP001072	1608548	Peru	Gastritis

### Ethics Statement

All patients involved have given informed consent for the samples to be used in historic or future studies. The consent was written, and ethics approval was obtained from the ethics committee of the Chinese Center for Disease Control and Prevention (China CDC) and the academic committee of the National Institute for Communicable Disease Control and Prevention, China CDC.

**Figure 1:**
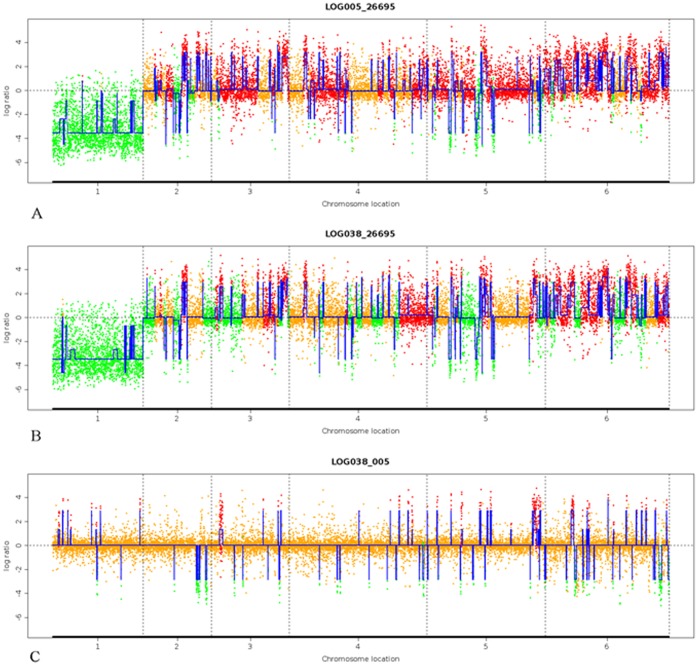
Comparison of HLJ005, HLJ038, and 26695 based on strain specific genes of the six sequenced genomes. A. Comparison between HLJ005 and 26695. B. Comparison between HLJ038 and 26695. C. Comparison between HLJ038 and HLJ005. Green spots show inferred loss while orange dots show no changes in the original data. Red spots show inferred increase. Segment 1 on the X-axis: probes of 26695 strain specific genes. Segment 2: probes of G27 strain specific genes. Segment 3: probes of HPAG1 strain specific genes. Segment 4: probes of J99 strain specific genes. Segment 5: probes of P12 strain specific genes. Segment 6: probes of Shi470 strain specific genes.

**Figure 2:**
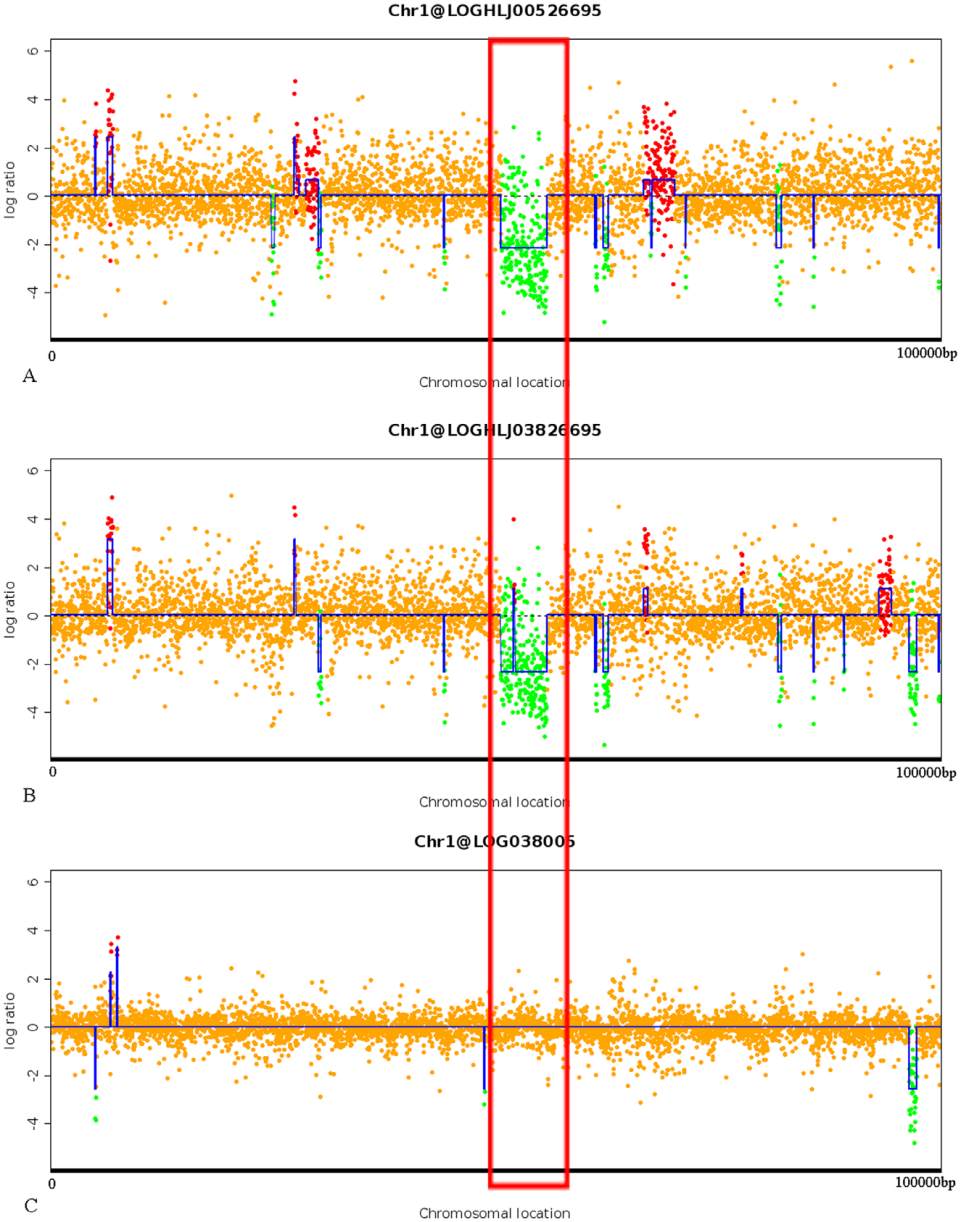
Comparison of HLJ005, HLJ038, and 26695 based on 0–100 kb of p12 genome. A. Comparison between HLJ005 and 26695. B. Comparison between HLJ038 and 26695. C. Comparison between HLJ038 and HLJ005. Green spots show inferred loss while orange dots show no changes in the original data. Red spots show inferred increase. The red rectangle indicates the predicted 5.2 kb absent region both in gastric cancer strain HLJ038 and HLJ005 based on the 0–100 kb of the reference P12 chromosome.

### DNA Preparation

Genomic DNA was extracted by using the DNeasy Blood & Tissue Kit (QIAGEN) according to the manufacturer’s instructions. The concentration of genomic DNA was adjusted to 120 ng/µl with nuclease-free water and to a volume of 40 µl. DNA was heated in a PCR machine (Biorad, USA) at 95°C for 10 min to obtain DNA fragments with sizes of 100 bp to 600 bp for genomic labeling. The results were visualized by 1% agarose gel electrophoresis.

**Figure 3:**
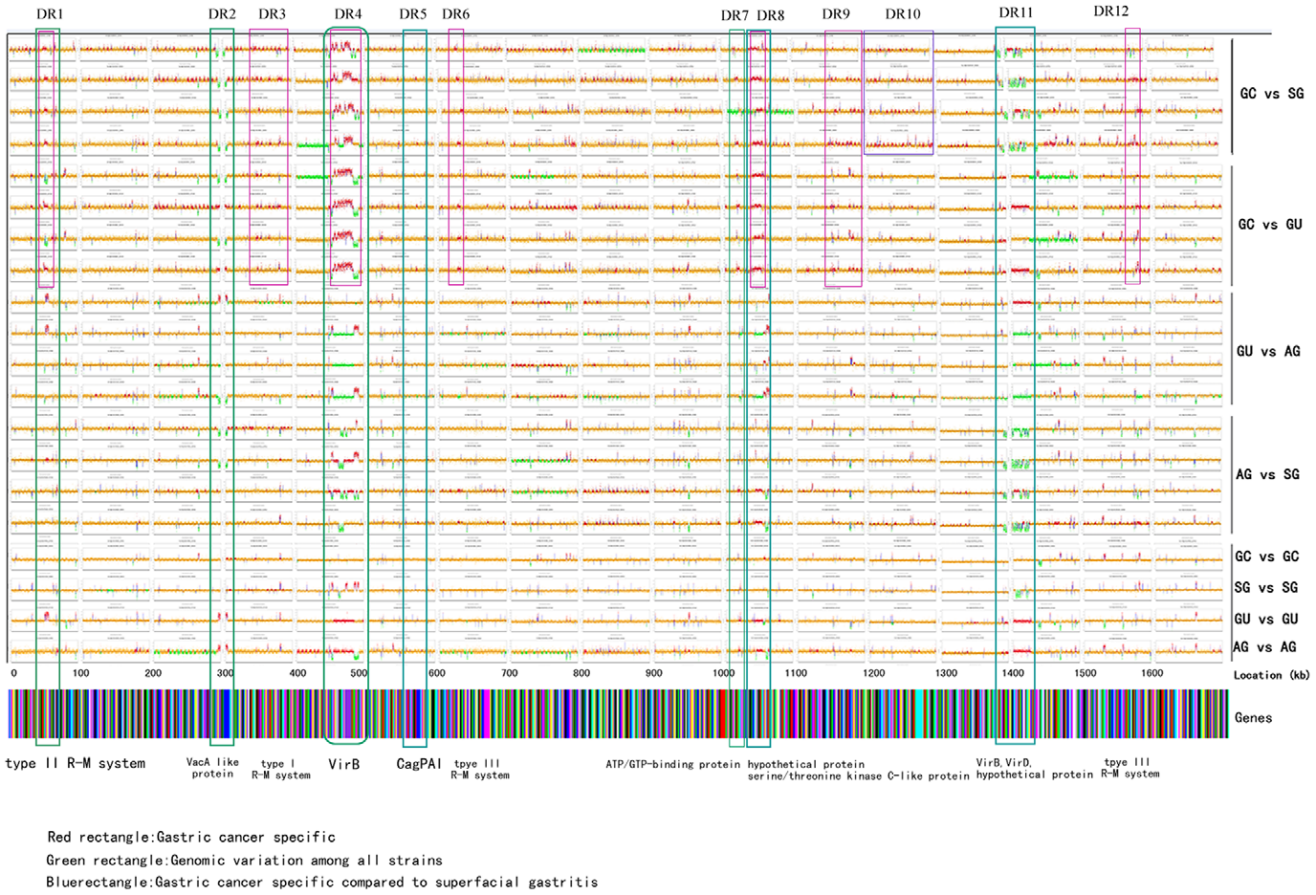
Genome comparison of eight *H. pylori* strains based on P12 reference sequence. Large variable regions were labeled with colored rectangles. Predicted gastric cancer strain specific regions were labeled with a red rectangle. General genomic variation among all strains was labeled with a green rectangle. A blue rectangle represents diverse regions obtained from comparison of gastric cancer strains and superficial gastritis strains. Corresponding coding proteins were briefly labeled at the bottom of each rectangle. GC vs SG denotes comparison between the two strains of gastric cancer and the two strains of superficial gastritis. DR, different region.

**Table 3:** Predicted variable genomic region based on reference genome P12.

DR	Gene	start	end	Gene description
DR1		49177	49869	adenine specific DNA methyltransferase
		49866	50933	cytosine specific DNA methyltransferase
		50930	52153	restriction endonuclease
		52297	53568	type II R-M system restriction endonuclease
		53568	56036	type II R-M system methyltransferase
DR2		295040	303760	vacuolating cytotoxin VacA-like protein
DR4	hsdS-1	448002	449183	type I R-M system S protein
	hsdM-1	449176	450807	type I R-M system M protein
		452423	453496	integrase/recombinase XercD family
		453578	454261	hypothetical protein
	virb6	454334	455632	VirB6 type IV secretion protein
		455598	455876	hypothetical protein
		455928	457328	hypothetical protein
		457332	457577	hypothetical protein
		457791	459272	hypothetical protein
		459405	460469	hypothetical protein
		460312	460803	hypothetical protein
		460810	461832	hypothetical protein
		462096	470522	DNA methylase
	parA	470785	471441	chromosome partitioning protein
		471523	471807	hypothetical protein
		471839	473017	hypothetical protein
	virD2-1	473042	474955	relaxase
		475253	475567	hypothetical protein
		475560	475841	hypothetical protein
	virD4-1	476105	477832	VirD4 coupling protein
		477879	478391	hypothetical protein
		478392	478682	hypothetical protein
		478679	479134	hypothetical protein
	virB11-1	479131	480072	VirB11 type IV secretion ATPase
		480072	480371	hypothetical protein
		480368	480631	hypothetical protein
		480624	480917	hypothetical protein
	virB10-1	480987	482252	VirB10 type IV secretion protein
	virB9-1	482252	483784	VirB9 type IV secretion protein
	virB8-1	483784	484953	VirB8 type IV secretion protein
	virB7-1	484957	485073	VirB7 type IV secretion protein
	virB3-1	489522	489788	VirB3 type IV secretion protein
	virB2-1	489789	490091	VirB2 type IV secretion protein
		490088	490372	hypothetical protein
		490433	491611	hypothetical protein
		491618	491911	hypothetical protein
		491931	492710	hypothetical protein
		494743	496638	hypothetical protein
		496664	497431	hypothetical protein
		497441	497791	integral membrane protein
	virb6	454334	455632	VirB6 type IV secretion protein
		455598	455876	hypothetical protein
		455928	457328	hypothetical protein
		457332	457577	hypothetical protein
DR6	res-1	628179	631121	type III R-M system restriction enzyme
DR8		1050977	1051564	serine/threonine kinase C-like protein
		1051604	1052053	serine/threonine kinase C-like protein
		1052213	1052728	serine/threonine phosphatase 2C-like
				protein
DR11	virB2-2	1396323	1396607	VirB2 type IV secretion protein
	virB3-2	1396619	1396882	VirB3 type IV secretion protein
		1396894	1397130	hypothetical protein
	virB4-2	1397130	1399706	VirB4 type IV secretion ATPase
	virB7-2	1399703	1399843	VirB7 type IV secretion protein
	virB8-2	1399836	1400972	VirB8 type IV secretion protein
	virB9-2	1400969	1402624	VirB9 type IV secretion protein
	virB10-2	1402591	1403829	VirB10 type IV secretion protein
		1403813	1405597	hypothetical protein
		1405501	1406055	hypothetical protein
		1406068	1407030	hypothetical protein
		1407047	1407322	hypothetical protein
	virB11-3	1407327	1408271	VirB11 type IV secretion ATPase
		1408268	1408786	hypothetical protein
	virD4-2	1408783	1411026	VirD4 coupling protein
		1413163	1413633	hypothetical protein
		1413603	1414406	hypothetical protein
		1414480	1415145	hypothetical protein
		1415123	1415401	hypothetical protein
		1415334	1415765	hypothetical protein
		1415750	1416160	hypothetical protein
		1416165	1416794	hypothetical protein
		1416897	1417178	hypothetical protein
		1417399	1417788	hypothetical protein
		1417796	1418086	hypothetical protein
		1418215	1418466	hypothetical protein
		1418394	1419248	hypothetical protein
		1421131	1421259	hypothetical protein
	virD2-2	1421785	1423818	relaxase
DR12		1575820	1579728	type IIS R-M system
				restriction/modification enzyme
		1580020	1582023	hypothetical protein
	res-4	1582157	1585066	type III R-M system restriction enzyme
	mod-5	1585069	1587111	type III R-M system methyltransferase

Note: DR, different region

### Microarray Design, Labeling, Hybridization and Stripping

A whole-genome CombiMatrix tiling CustomArray™ 90 K (Mukilteo, WA, USA) was used in this study. Combimatrix is a highly flexible platform that allows for the synthesis of in situ oligos (35mers) through the use of an electrochemistry-based method. This innovative technology also allows us to reuse the same microarray several times. Traditional cDNA microarrays involve large amounts of primer design and PCR for preparation of amplicon probes to be used for microarray printing. This process can be cumbersome and time consuming. Compared to all other platforms, including cDNA microarray, the Combimatrix drastically reduces fabrication and analytic costs. Thus, this process is more rapid and convenient. The platform has established a complete protocol for genomic analyses from sample labeling to data extraction [39]–[41].The array contained 90000 *in* *situ* synthesized oligonucleotide probes. In total, six sequenced genomes were taken for analysis. Genomic information for each strain is summarized in Table 2. Genome alignments were performed by Combimatrix to obtain strain specific genes of each sequenced strain. We then took the whole genome of P12 as the reference sequence for tiling designation, which occupied about 70000 probes on the 90 k array. The sequences were first broken up into 100,000 bp blocks. We designed all possible probes for every 19 bp and selected the best one for each interval. At each position, a probe was extended until the Tm reached the desired Tm. Probes were then tested for quality, and the best probe (probe length 35∼40 mer) was chosen. Both strain-specific genes and plasmid genes were then chosen for tiling for the remaining 20000 probes based on the same criteria.

**Figure 4:**
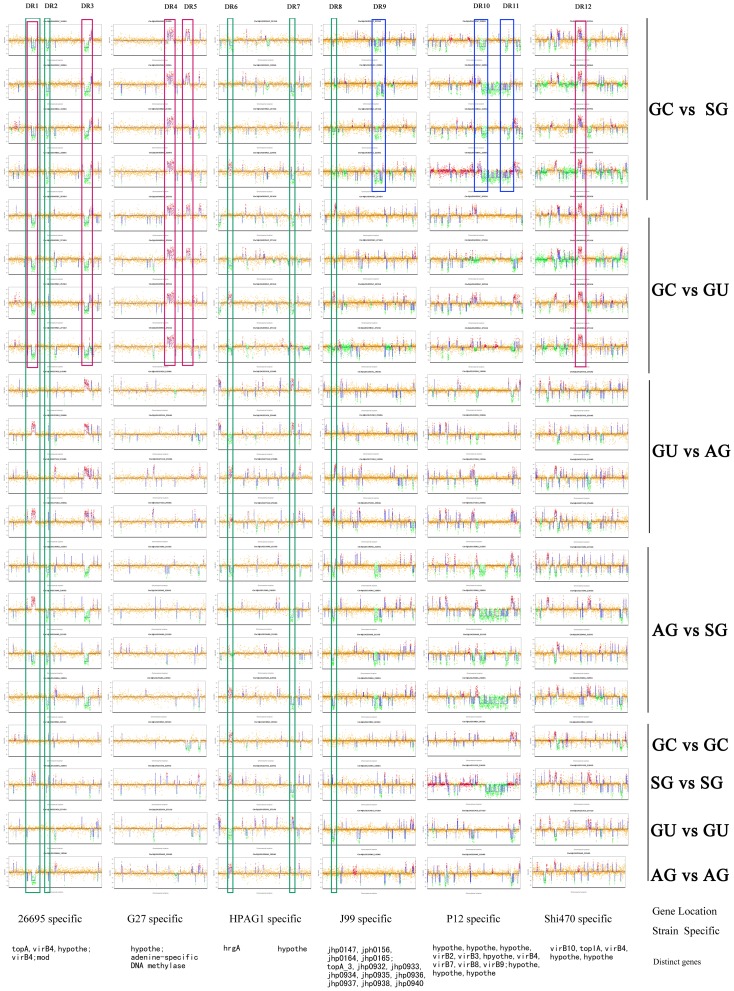
Comparison of eight *H. pylori* strains based on strain specific genes of six sequenced strains. Large variable regions were labeled with colored rectangles. Predicted gastric cancer strain specific regions were labeled with a red rectangle. General genomic variations among all strains were labeled with a green rectangle. A blue rectangle represents diverse regions obtained from comparison of gastric cancer strains and superficial gastritis strains. Corresponding coding proteins were briefly labeled at the bottom of each rectangle. GC vs SG denotes comparison between the two strains of gastric cancer and the two strains of superficial gastritis.

**Table 4:** Predicted variable genes based on strain specific genes of the six sequenced strains.

Strain	DR	Gene	start	end	Gene description
	DR1	HP0440	457297	459330	DNA topoisomerase I (topA)
		HP0441	459333	459333	VirB4 homolog
26695		HP0442	461749	462015	hypothetical protein
		HP0443	462016	462318	hypothetical protein
strain		HP0444	462315	461756	hypothetical protein
		HP0445	463954	464139	hypothetical protein
specific	DR2	HP0456	475056	475508	hypothetical protein
		HP0457	475826	476089	hypothetical protein
genes		HP0458	476101	476337	hypothetical protein
		HP0459	476337	478913	virB4 homolog (virB4)
		HP0460	479043	479531	hypothetical protein
		HP0461	479557	479649	hypothetical protein
		HP0462	480062	481159	type I restriction enzyme S protein (hsdS)
		HP1366	1427688	1428959	type IIS restriction enzyme R protein (MBOIIR)
	DR3	HP1367	1428975	1429757	type IIS restriction enzyme M1 protein (mod)
		HP1368	1429744	1430607	type IIS restriction enzyme M2 protein (mod)
G27	DR4		1046540	1048216	hypothetical protein
			1048432	1048734	hypothetical protein
strain			1053564	1054550	competence protein
			1076345	1077382	hypothetical protein
specific			1081055	1082440	hypothetical protein
			1082440	1083642	hypothetical protein
genes			1083705	1084781	integrase-recombinase
					protein
	DR5		1351624	1352001	adenine-specific DNA methylase
			1419432	1420331	adenine-specific DNA methylase
HPAG1	DR6	HrgA	94738	95736	HrgA
strain	DR7		1410157	1412718	hypothetical protein
Specific genes					
J99	DR8	jhp0164	178219	179565	putative restriction enzyme
strain		jhp0165	179558	180778	hypothetical protein
Specific genes	DR9	jhp0929	1032025	1032477	hypothetical protein
		jhp0930	1032591	1032833	hypothetical protein
		topA_3	1032846	1034906	topoisomerase I
		jhp0932	1034961	1035431	hypothetical protein
		jhp0933	1035401	1036204	hypothetical protein
		jhp0934	1036277	1037296	hypothetical protein
		jhp0935	1037343	1037885	hypothetical protein
		jhp0936	1038083	1038616	hypothetical protein
		jhp0937	1038613	1039878	hypothetical protein
P12	DR10	virB2-2	1396323	1396607	VirB2 type IV secretion protein
strain		virB3-2	1396619	1396882	VirB3 type IV secretion protein
Specific genes			1396894	1397130	hypothetical protein
		virB4-2	1397130	1399706	VirB4 type IV secretion ATPase
		virB7-2	1399703	1399843	VirB7 type IV secretion protein
		virB8-2	1399836	1400972	VirB8 type IV secretion protein
		virB9-2	1400969	1402624	VirB9 type IV secretion protein
		virB10-2	1402591	1403829	VirB10 type IV secretion protein
			1403813	1405597	hypothetical protein
			1405501	1406055	hypothetical protein
			1406068	1407030	hypothetical protein
			1407047	1407322	hypothetical protein
		virB11-3	1407327	1408271	VirB11 type IV secretion ATPase
			1408268	1408786	hypothetical protein
		virD4-2	1408783	1411026	VirD4 coupling protein
			1413163	1413633	hypothetical protein
			1413603	1414406	hypothetical protein
			1414480	1415145	hypothetical protein
			1415123	1415401	hypothetical protein
			1415334	1415765	hypothetical protein
			1415750	1416160	hypothetical protein
			1416165	1416794	hypothetical protein
			1416897	1417178	hypothetical protein
			1417399	1417788	hypothetical protein
			1417796	1418086	hypothetical protein
			1418215	1418466	hypothetical protein
			1418394	1419248	hypothetical protein
			1421131	1421259	hypothetical protein
		virD2-2	1421785	1423818	relaxase
	DR11		1466136	1466765	hypothetical protein
			1470038	1471000	type II R-M system restriction endonuclease
			1470981	1471751	type II R-M system restriction endonuclease
			1479129	1479917	hypothetical protein
			1486667	1487452	hypothetical protein
			1487452	1489119	hypothetical protein
			1492159	1492494	hypothetical protein
			1507434	1507625	hypothetical protein
			1524027	1526738	DNA polymerase I
			1528042	1530078	type IIS R-M system methyltransferase
			1555145	1556065	hypothetical protein
Shi470	DR12		874998	876074	integrase/recombinase (xerD)
strain			876139	877341	hypothetical protein
Specific genes			877341	878726	hypothetical protein
			880915	882183	hypothetical protein
			882184	883221	hypothetical protein
			883375	891786	hypothetical protein
			902243	903478	ComB3 protein
			906132	908066	topoisomerase I
			908059	910557	DNA transfer protein
			910570	910830	hypothetical protein
			910831	911133	hypothetical protein
			911758	913038	hypothetical protein
			913049	913813	hypothetical protein
			913835	915454	hypothetical protein

Note: DR, different region.

Four micrograms of heated DNA from each *H. pylori* strain was labeled with Cy5-ULS using the Kreatech ULS array CGH Labeling kit (EA-005, Kreatech, Netherlands) according to the manufacturer’s instructions and then hybridized to the microarray. Experiments were performed according to CombiMatrix protocol. Microarrays were pre-hybridized with 6×SSPE containing 0.05% Tween-20, 5×Denhardt’s solution, and 100 ng salmon sperm DNA for 30 min at 50°C. The Cy5-ULS labeled DNA fragments were then hybridized in the hybridizing solution (6×SSPE and 0.05% SDS) by denaturing at 95°C for 3 min and then incubating for 16 h at 50°C. Post-hybridization wash steps were 6×SSPET for 5 min at 50°C, 3×SSPET for 1 min, 0.5×SSPET for 1 min, and PBST for 1 min at room temperature. After hybridization and imaging, the microarray was stripped using CustomArray™ Stripping Solution. The well known sequenced strain 26695 and an additional eight strains from Heilongjiang province were hybridized to the chips.

**Figure 5:**
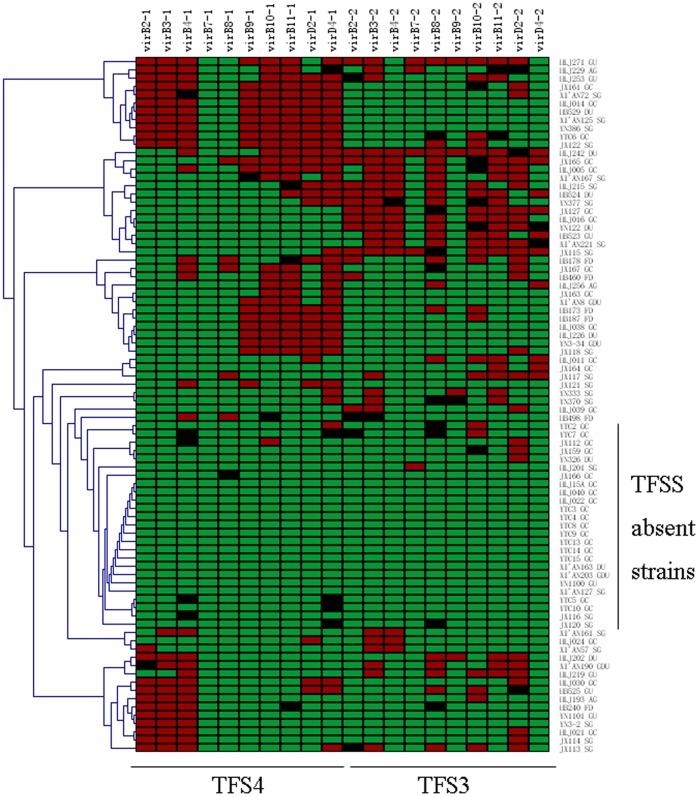
Gene content analysis of TFS4 and TFS3 in eighty-four *H. pylori* isolates by PCR. The presence of individual genes is indicated in red, and their absence in green. Non-specific amplicon is shown in black.

### Microarray Scanning and Statistical Analysis

Hybridized microarrays were covered with imaging solution and scanned with an Axon GenePix™ 4000B. All of the nine hybridizations were scanned at the same PMT value. The stripped microarray was also scanned with the same PMT gain value (475) to evaluate background noise as well as the stripping efficiency. Data was extracted by using Microarray_Imager_5.9.3 software. The foreground median of 635-nm signal intensities of each spot was used for the subsequent analysis. Genes that had at least five contiguous oligonucleotides with a minimum 635-nm intensity of 800 intensity units, and had at least one tile with 635-nm intensity >1200 intensity units were defined as positive. We defined background signal intensity as the median signal intensity of all spots locations that did not have any oligos. QC-oligoB-AS-3 spots were also included in the background. QC-oligoB-AS-3 spots were CombiMatrix quality control oligonucleotides that occur 459 times on the array. Since QC-oligoB-AS-3 does not align to the *H. pylori* genome, these spots could be considered for background calculation for further analysis. On each of the arrays, the background signal was <400 intensity units, indicating that our signal cutoff of 800 was sufficiently above background for identification. Based on the above described criteria for gene identification, information of probe location and corresponding hybridization signals were extracted for further analysis. For ease of analysis, all genes were separated into two independent files, one including genes located in the *H. pylori* P12 genome and another including strain specific genes in each of the other strains, which are named chromosome 1–6 (this also included P12 strain specific genes). Isolates of gastric cancer were represented as GC, isolates of gastric ulcers were represented as GU, isolates of chronic superficial gastritis were represented as SG, and isolates of atrophic gastritis were represented as AG. Signal comparison was performed between the two strains in GC, GU, SG, and AG. Comparison was also performed by GC vs GU, GU vs AG, GC vs SG, and AG vs SG. Signal ratios of two hybridized strains were log2 transformed and imported into a web tool ADaCGH, which was used for the analysis of microarray datasets to show gains and losses of genes between the two genomes. The data was centered and segmented in ADaCGH by employing a position-dependent segmentation algorithm CBS (circular binary segmentation) to partition data points into absent, present, and no change sequence segments [42], [43]. Chi-square was used to analyze the association of genes with disease by comparing one disease group (i.e., GC) with the other two groups (i.e., SG and GU), as well as to analyze the TFSS gene distributions among different disease groups. P value <0.05 was considered significant.

**Table 5:** Distribution of *hrg*A in different diseases.

Diseases	hrgA		Total
	+	−	
GC	25	8	33
GU	5	2	7
DU	4	4	8
GDU	0	4	4
SG	9	14	23
AG	1	2	3
FD	3	3	6
Total	47	37	84

Note: GC, gastric cancer. GU, gastric ulcer. AG, atrophic gastritis, DU, duodenal ulcer GDU, gastroduodenal ulcer, FD, functional dyspepsia, SG, non-atrophic gastritis.

### Microarray Data Deposition

MIAME compliant microarray data of this study has been deposited at the Gene Expression Omnibus database (GEO, http://www.ncbi.nlm.nih.gov/geo/) under series accession number GSE32107.

### Validation of genes associated with diseases by PCR

The results for some variable genes found to be associated with diseases by microarray analysis were confirmed using gene-specific PCR. We preliminarily selected 25 such genes involved in two TFSS systems, a predicted 5.2 kb variable region, and an *hrgA* gene, which was reported to have a high occurrence rate in strains isolated from gastric cancer patients in East Asia. Specific primers were selected based on the sequences of these genes. To investigate the distribution of genes that were predicted as genomic distinctive regions, eighty-four strains were selected for PCR analysis of the 25 genes. A heatmap of type IV systems genes was created using Mev_4_0-2.

## Results

### Microarray Hybridization and Statistical Analysis

In order to test whether the established *H. pylori* microarray analysis system can accurately predict genomic distinction, we first analyzed strains 26695, HLJ005, and HLJ038 based on strain specific genes and 0–100 kb of the reference P12 genome sequence. As shown in Figure 1, six segments on the X-axis represent strain specific genes of the six sequenced genomes. Probes of segment one are strain specific genes of 26695. When comparing the hybridization signal of gastric cancer strain HLJ005 to 26695 with the web tool ADaCGH, almost all of these genes were predicted to be absent in HLJ005 (Figure 1 A). The same pattern was found in another gastric cancer strain HLJ038 (Figure 1 B).When comparing HLJ005 and HLJ038, only some minor variable probes were found in the six segments (Figure 1 C). These analyses indicate that this custom designed microarray platform accurately predicts gene absence or presence in different clinical *H. pylori* isolates. This was further validated by PCR to confirm a predicted 5.2 kb absent region both in gastric cancer strain HLJ038 and HLJ005 based on the 0–100 kb of the reference P12 chromosome (Figure 2).

Variable genes of the tested strains were first obtained based on the reference P12 genome sequence (Figure 3, Table 3). Twelve genomic regions with the most variable tiles among the eight strains are marked by a rectangle. Predicted gastric cancer strain specific regions were labeled with a red rectangle according to the comparison of GC vs SG and GC vs GU. Seven such areas were acquired, and encoded proteins were listed in Table 3 (DR1, 3, 4, 6, 8, 9, 12). The number of these variable genes present in the group of gastric cancer strains was statistically higher than those for the GU and SG strains (P<0.05). These regions mainly coded for proteins related to the type I R-M system, type II R-M system, type III R-M system, TFSS system, and hypothetical proteins. General genomic variations among all strains were labeled with a green rectangle. Seven such regions were identified (DR 1, 2, 4, 5, 7, 8, 11). No significant correlation was found for the unique absence or presence of TFS4 or TFS3. However, absence of both TFS3 and TFS4 system genes display significant variation comparing GC strains to GU, DU, or SG strains (P<0.05). Among the 25 strains that are absent for these two systems, 17 strains are GC strains (68%). No significant variation was found for the association of any gene cluster with special geography. In addition, cagPAI was also labeled with a green rectangle for its diversity among reported strains (DR5). However, cagPAI were conserved among the tested strains of this study. The blue rectangle represents diverse regions obtained from comparison of gastric cancer strain with superficial gastritis strain (DR10). Some of the corresponding coding proteins were briefly labeled at the bottom of each rectangle. GC vs SG denotes comparison among the two strains of gastric cancer and the two strains of superficial gastritis.

Comparison of eight *H. pylori* strains based on strain specific genes of six sequenced strains is shown in Figure 4. Twelve large variable regions are labeled with rectangles, including five red rectangles denoting predicted gastric cancer strain specific regions (DR1, 3, 4, 5, 12), three blue rectangles denoting diverse regions obtained from comparison of gastric cancer strains and superficial gastritis strains (DR 9, 10, 11), and five green rectangles denoting common variable genes among all strains (DR 1, 2, 6, 7, 8). These predicted variable genes based on strain specific genes of the six sequenced strains were listed in Table 4.

### Gene Variation Confirmed by PCR

To further validate the microarray results, we performed PCR to confirm a predicted 5.2 kb absent region both in gastric cancer strain HLJ038 and HLJ005 based on the 0–100 kb of the reference P12 chromosome (Figure 2 A B). PCR results validated that the 5.2 kb region is absent in the two gastric cancer strains but present in 26695 (data not shown). As shown in Figure 2C, comparing 0–100 kb of HLJ005 and HLJ038 shows minor differences between the two GC strains. These scattered differing genes are suspected to be strain-specific genes of HLJ005 and HLJ038.

A heatmap of variable genes detected among the three *H. pylori* test strains was also confirmed using PCR. Green denotes absence of genes; red indicates presence of genes. The distinct regions of the three test strains were clustered with a dendrogram, which was constructed by hierarchical analysis of the presence or absence of genes. Amplification of some genes showing a weak PCR product band or a band of incorrect size are represented in black. One possible explanation for this is that sequence variation in these genes occurs in the primer binding region. The twenty variable genes of the TFSS3 and TFSS4 in the eight GC isolates observed by ADaCGH were confirmed by PCR, showing concordance with the microarray data. The prevalence of these genes in isolates from 76 additional patients was tested by PCR (Figure 5). These genes were found to be absent in 18 of 33 GC strains (55%). Other strains harbor two or more of these gene. These data support the association of the loss of these genes in isolates from GC patients.

## Discussion

In the eight Chinese isolates studied by microarray, we found 14 significant variable regions when comparing GC isolates to SG or GU isolates based on the P12 reference genome (Figure 3, Table 2). These regions mainly coded for proteins related to the type I R-M system (DR3), type II R-M system (DR1), type III R-M system (DR6, DR12), TFSS system (DR4, DR11), and hypothetical proteins (DR8). These findings are consistent to those of previous reports. Previous research found a remarkable variety of restriction-modification (R-M) systems in *H. pylori*. Bacteria utilize R-M systems through conjugative plasmids or bacteriophages as a defense against invasion by foreign DNA. Recently, restriction–modification systems have been reported to be associated with *H. pylori* virulence. The best understood R-M system is the type II R-M family. Type II R-M systems include two enymes: one is a restriction endonuclease which cleaves DNA within a specific 4–8-bp sequence, while another is a methyltransferase which can specifically methylate DNA at adenine or cytosine residues. Bacteria can protect DNA from cleavage through this mechanism [44]–[48]. We identified a number of variable genes involved in these restriction-modification systems among the microarray tested strains. The results indicate a strong correlation between *H. pylori* R-M system and pathogenesis of gastric cancer. Further investigations involved in functional analysis of the R-M system are very necessary to demonstrate the potential mechanism.

A previous Chinese study, performed by Han, Y. H. et al, also analyzed the genomic variation of *H. pylori* isolates from patients with gastroduodenal diseases [34]. They found two diverse regions in the *H. pylori* genome that corresponded to plasticity zones (PZ) 1 and 2. Our results are partially consistent with one diverse region of their findings. This region comprises genes from HP0424 to HP0462. However in our study, we only identified genes in these regions from HP0440 to HP0445 and HP0456 to HP0462. For diverse region 2 from HP0984 to HP1009, we have not observed wide variation among our strains except for a minor difference of HP0993 and HP0994, which are predicted to be absent in GC strains HLJ005 and HLJ038 compared to the SG strain HLJ220. In addition to these distinctions, we found the other diverse region among strains based on 26695 strain specific genes. They are from HP1366 to HP1368 and encode type IIS restriction enzyme related proteins. For the other genes that were found by Han, Y. H. et al, such as HP0447, HP0704, and jhp0918, they claimed that certain genotypes had higher prevalence of these genes in DU or GC groups than in CG groups, but this was not inspected in our microarray that studied eight Heilongjiang province strains. These discrepancies may be due to the differences in strain selection. It is also possible that technical differences contribute to the discrepancies observed. Another previous comparative genomic study reported that genes located in the plasticity zones such as jhp0947 and jhp0949 are associated with disease [49]–[51]. For these genes, we also detected a small scattered variable region among the eight microarray tested strains. But we have not performed PCR tests in more strains for these genes. In the future, we will follow this work up with systematic analysis. Despite these variable genes described above, we found that the *cag* PAI was significantly conserved in the eight tested isolates from Heilongjiang province, which is consistent with the fact that *cag* PAI is geographically diverse.

The distribution of genes of type-IV secretion systems in Chinese isolates were also investigated in detail. *H. pylori* have at least three type-IV secretion systems (TFSS). The first type IV secretion system gene cluster is located in the cag pathogenicity island, which mediates the injection of the toxin CagA and is thought to be the most important apparatus that contributes to pathogenesis. Several studies have described and discussed the role of the cag-pathogenicity island encoded type IV secretion system in *H. pylori* pathogenesis [9]–[11]. However, the potential mechanism for *cag* PAI induced cancer is still under known. The second gene cluster is involved in a *comB* locus that confers competence for DNA transformation [52]. Recently, a third TFSS responsible for DNA conjugation has been demonstrated and discussed in various studies. This TFSS system helps *H. pylori* rapidly acquire new genetic features and adapt to changes in the environment [53]–[56]. We explored genes involving two such TFSS systems of P12 in our study. Consistent with previous studies, many genes in these systems are variably present (Figure 5). Although complete TFS3 or TFS4 type IV secretion systems were found in some strains (i.e., HLJ242), partial absence of type IV secretion system genes seem to be more common. None of the tested strains harbored the two TFSS system genes despite a possible failure to amplify *virB7-1*. All of our tested strains did not contain *virB7-1*; this is probably because the length of *virB7-1* is only 117 bp. Therefore, it is difficult to design a pair of primers that efficiently amplify this gene target. Although no significant correlation was found for the unique absence or presence of TFS4 or TFS3, absence of both TFS3 and TFS4 system genes displayed significant variation comparing GC strains to GU, DU, or SG strains (P<0.05). It is intriguing that large proportions of GC strains in our study lost these two systems, which are reported to be involved in the transfer of genomic fragments and the restriction-modification system through a special mechanism. More thorough research is required to explore the potential function or mechanism for losing TFSS systems in GC isolates.

We also found one of the four genes with the 5.2 kb predicted variable segment, a gene encoding cytosine specific DNA methyltransferase, is present in 11 of 12 GC strains isolated from Shandong province (represented as YTC). This suggests that special genomic characteristics may exist in this group of strains. Based on the strain specific genes of the six sequenced genomes, several variable genes were identified including an *hrgA* gene (Figure 4, Table 3, DR6), which has been previously reported to be a strain-specific gene that might be associated with gastric cancer among *H. pylori* isolates from Asian patients [57]. For the distribution of *hrg*A in our 33 tested gastric cancer strains, 25 (76%) tested positive for the presence of this gene, which was higher than previously reported rates of prevalence (43%). 21 of the remaining 51 (41%) isolates of non-cancer patients were positive for the gene. There is no statistical significance for the distribution of hrgA in different diseases (Table 5). There are many additional variable genes that were screened by microarray among the tested strains. Validating each of these genes one by one by PCR would be laborious and be contrary to the goals of a microarray-based experiment, which is rapid and high throughput genomic analysis. Since the custom HP 90 k tiling microarray has been well designed, established, and evaluated in this study, a greater number of strains need to be analyzed by this tool in order to gather more detailed and reliable information about which variable genes contribute to different disease status.

We initially tried to cover all sequenced strains for probe design. At the time, only six strains had completed genome maps online. P12 was selected as a major tiling reference sequence since it is the longest. Strain specific genes of an additional five strains were also tiled to search for possible differences among the tested strains. To quickly evaluate the accuracy of this array design, we initially hybridized a well known sequenced strain 26695 and two strains isolated from two gastric cancer patients in Heilongjiang province. Paired comparison of these strains for a 0–100 kb region based on the P12 reference genome revealed several regions with genetic variation. Specific primers were designed for these genes, and PCR validation was performed in 26695, HLJ005, and HLJ038. Of the 12 genes tested, 11 were consistent with the microarray prediction(data not shown). For the strain specific genes of the six sequenced strains, our results precisely predicted that all strain specific genes of 26695 are actually present in 26695 compared to HLJ005 and HLJ038. These results indicate that the designed microarray can provide an accurate analysis for exploring genomic diversity among different HP strains.

The aim of this study was to search for genomic characteristics that may contribute to various digestive diseases. Strain selection is one of the most important aspects of this study because genomic diversity exists among strains of different geographic origin. To eliminate this interference, we selected eight strains that originated from a northeast province of China. Heilongjiang province has one of the highest incidence rates of gastric cancer. Our results show that comparison of the strains with the same diseases (HLJ005 vs HLJ038) have less variation than the strains with different diseases, which indicates that it is reasonable to further investigate more paired strains with different clinical diseases.

With the rapid development of next generation sequencing technology and reduced costs, microarrays will be completely replaced by sequencing in the future. However, no one can predict how long this change will take. We believe, that because of its low cost, microarray technology will continue to play an important role in genomic and expression level analyses of bacterial pathogens for several years. It also has easy handling and rapid time saving advantages. The established in situ synthesized high density oligo tiling microarray was successfully utilized in this study and can be used as a high throughput analytic tool for comparative genomics of *H. pylori*. Results of our study promote further understanding of specific disease-associated genes that can serve as novel biomarkers for identification of gastroduodenal diseases.
